# A Prospective, Multi‐Center, Clinical Trial of a 10‐kHz Spinal Cord Stimulation System in the Treatment of Chronic Pelvic Pain

**DOI:** 10.1111/papr.12932

**Published:** 2020-08-08

**Authors:** Jordan L. Tate, Thomas Stauss, Sean Li, Anand Rotte, Jeyakumar Subbaroyan

**Affiliations:** ^1^ Alliance Spine and Pain Centers Canton Georgia U.S.A; ^2^ Advanced Pain Management Milwaukee Wisconsin U.S.A; ^3^ Premier Pain Centers Shrewsbury New Jersey U.S.A; ^4^ Nevro Corp Redwood City California U.S.A

**Keywords:** 10‐kHz SCS, chronic pelvic pain, visual analog scale

## Abstract

**Background:**

Chronic pelvic pain (CPP) is a debilitating condition that often leads to disability and does not respond to conventional treatments. This study was conducted to evaluate the effects of paresthesia‐independent 10‐kHz spinal cord stimulation (SCS) in subjects with CPP.

**Methods:**

This prospective, single‐arm pilot study enrolled subjects with clinical diagnoses of CPP and mean pain scores of ≥ 5.0 cm on a 10‐cm VAS. Subjects underwent trial stimulations with 10‐kHz SCS, and those who had successful trial stimulations (≥40% pain relief) received permanently implanted devices and were followed for 12 months.

**Results:**

Of the 21 subjects who underwent the 10‐kHz SCS trial, 17 were successful and 14 subjects received permanent implants. No neurological deficits were observed in any subjects and all adverse events (AEs) were resolved without sequelae during the study period. Over 12 months, mean VAS scores decreased by 72% from baseline, and 10 of 13 subjects (77%) were responders (≥50% pain relief). Pain remission (VAS score ≤ 3.0 cm) was reported by 8 of 13 subjects (62%), and mean pain scores on the short‐form McGill Pain Questionnaire 2 decreased as well. Pain Disability Index scores declined by 29 points, and 85% of the subjects reported satisfaction.

**Conclusions:**

Paresthesia‐independent stimulation with 10‐kHz SCS reduced pelvic pain in subjects with CPP and was not associated with any unexpected AEs. While larger, controlled studies are needed, results of this study suggest that this therapeutic modality could potentially treat patients with CPP while improving their quality of life.

## Introduction

Chronic pelvic pain (CPP) is characterized by continuous or intermittent pain in the lower abdomen or pelvis lasting at least 6 months that impairs patient functioning and quality of life and can be cyclical or noncyclical in nature.^1^ CPP is a relatively common condition caused by a variety of complex disorders such as endometriosis, pudendal neuralgia, coccydynia, prostadynia, vulvodynia, and painful bladder syndrome.^2^ Population surveys have reported a prevalence in adult women of about 15% in both the United Kingdom^3^ and United States.^4^ CPP is less prevalent in men and occurs most often due to chronic prostatitis/chronic pelvic pain syndrome and interstitial cystitis/bladder pain syndrome, which have a worldwide prevalence in males of 2% to 16%.^5^ Because CPP is a complex disorder in etiology and presentation, the treatment of CPP is challenging and hampered by the existence of few effective therapeutic options.

Pharmacologic treatments are the primary clinical intervention used for CPP and include tricyclic antidepressants, anticonvulsants, opioids, muscle relaxants, antibiotics, α‐blockers, and pentosan polysulfate.^6^ However, there is little evidence showing these agents are superior to placebo, and many are associated with undesirable side effects. Surgery can also be used to address some cases of CPP, but there has been a decrease in emphasis on invasive interventions and increased attention on alternatives such as physical therapy and neuromodulation.

Patients with CPP whose pain does not respond to medications require more aggressive interventions such as nerve blocks, which consist of injecting therapeutic agents such as local anesthetics and or steroids under ultrasound or other imaging guidance.^7^ Nerve blocks can be used both diagnostically to identify the affected nerves as well as therapeutically to relieve pain symptoms. Pudendal nerve blocks are used for both diagnostic and first‐line treatment for pudendal neuralgia, while ganglion impar blocks can be effective for perineal pain and coccydynia, and superior hypogastric blocks have been used to treat various pelvic pain types such as severe penile pain.^2^, ^8^ However, in many cases nerve blocks provide only temporary relief from symptoms.

Spinal cord stimulation (SCS) has been shown to have a positive effect in patients with CPP in a limited number of case reports.^9^ However, the various dermatomes of the pelvic region span a relatively large distance on the spinal cord, and the optimal placement of the epidural leads for administering SCS is difficult to determine.^2^, ^10^ Conventional SCS therapy requires the overlapping of paresthesia and the area of chronic pain. The complexities of the pelvic sensory neuroanatomy and the clinical requirements of paresthesia‐based SCS have limited the scope of SCS therapy in treating CPP.^6^

High‐frequency SCS delivered at a frequency of 10 kHz has been shown to be superior to conventional SCS for treating low back and leg pain,^11^, ^12^ and a limited case series has shown that this method can be effective in patients with CPP as well.^13^ This study was conducted in order to better evaluate the safety and effectiveness of 10‐kHz SCS for treating patients with CPP.

## Methods

This was a single‐arm, prospective, multicenter, postmarket, pilot study designed to evaluate the safety and effectiveness of 10‐kHz SCS in subjects with CPP. Subjects were enrolled at 3 sites in the United States. The investigational plan, amendments, and informed consent forms were reviewed and approved by the Western Institutional Review Board before implementation, and the study complied fully with the U.S. Code of Federal Regulations and recommendations guiding physicians in biomedical research by the 18th World Medical Assembly, Helsinki, Finland.

The eligibility of consenting subjects was determined based on pragmatic inclusion and exclusion criteria practiced in interventional pain clinics as standards of care (Tables S1 and S2). Major inclusion criteria included having a clinical diagnosis of CPP, a mean pelvic pain score ≥ 5.0 cm on the VAS, and pain that was refractory to conventional medical treatment for ≥3 months. Important exclusion criteria included mechanical spine instability; clinically significant spinal stenosis, objective evidence of epidural scarring, and/or any signs or symptoms of myelopathy; and previous use of a neuromodulation device.

### Procedures

**Figure 1:**
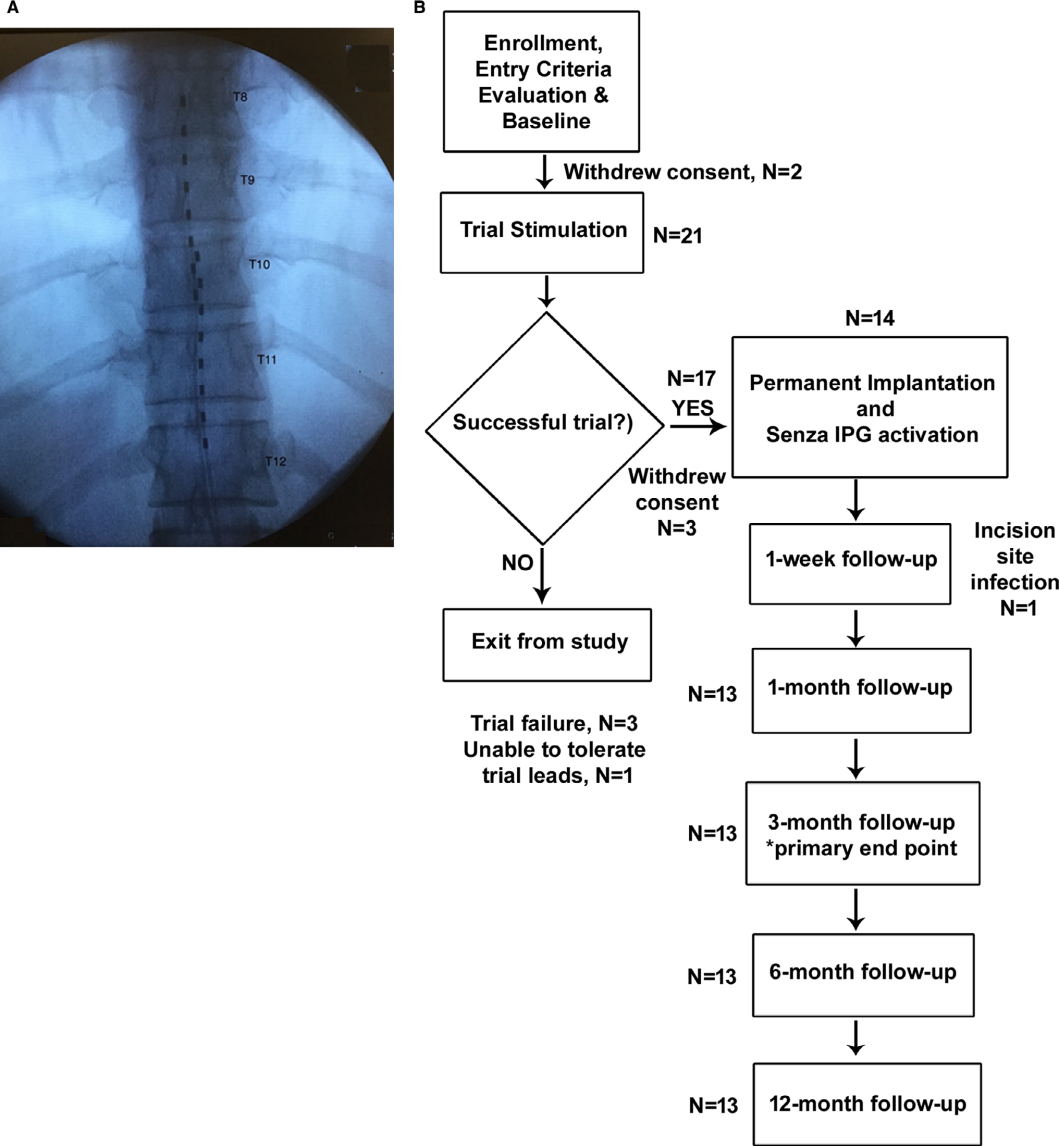
A, Representative x‐ray showing epidural lead placement spanning vertebral levels T8 through T12. B, Study design. IPG, implanted pulse generator.

Eligible subjects who met all inclusion criteria and none of the exclusion criteria underwent implantation with epidural leads spanning the T8 to T12 vertebral segments and underwent a temporary trial stimulation with 10‐kHz SCS (SENZA; Nevro Corp., Redwood City, CA, U.S.A.). The leads were positioned to span vertebral levels T8 to T12 (Figure 1A), and the trial stimulation lasted up to 7 days. Trial stimulations resulting in ≥40% reduction in pelvic pain, as assessed by VAS scores, were deemed successful, and those subjects were eligible to receive a permanently implanted 10‐kHz SCS system. The 40% pain reduction threshold that defined trial responses was lower than the 50% threshold used to define responders for the primary endpoint of pain. This is to avoid screening genuine responders during the brief trial period.^11^, ^14^

Subjectings who received permanent implants were stimulated at a frequency of 10‐kHz, a pulse width of 30 microseconds, and amplitudes individually adjusted to maximize pain relief. Programming adjustments were offered, if necessary, at follow‐up visits after 3, 6, 9, and 12 months of stimulation.

### Outcome Assessments

Outcome measures used to assess pelvic pain included subject‐reported VAS scores on a 10‐cm scale and the short‐form McGill Pain Questionnaire 2 (SF‐MPQ‐2),^15^ which was used to determine the severity and characteristics of subjects’ pain. The Pain Disability Index (PDI)^16^ was used to evaluate functional impairment, while the Global Impression of Change (GIC) scale^17^ was used to quantify subjects’ and investigators’ perception of functional change after the initiation of stimulation. The safety of 10‐kHz SCS was assessed in these subjects using tests of neurological functioning (including motor, sensory, and reflex functions) administered at baseline and follow‐up visits, and the investigators also reported all adverse events (AEs).

### Statistics

All continuous variables are presented as means ± standard error of the mean (SEM) intervals, as appropriate. Safety analysis was performed in all subjects who were trialed with a 10‐kHz SCS system, and efficacy analyses were performed in subjects who received permanent implants and had primary endpoint assessment. For endpoint analysis, responders were defined as subjects who reported pain relief of ≥50%, a threshold used in previously published studies of 10‐kHz SCS therapy,^11^, ^14^ and pain remission was defined as pain intensities of VAS ≤ 3.0 cm.^18^

## Results

The study enrolled 23 subjects, and their demographic and clinical characteristics are summarized in Table 1. Most subjects were female (91%). This group had a mean time since diagnosis of 9.7 years and a mean baseline VAS score of 8.0 cm. The etiologies of CPP among this population were diverse (Figure S1), including post‐surgical complications, postpartum complications, and interstitial cystitis/painful bladder syndrome in more than a quarter of the cases.

**Table 1:** Demographics and Clinical Characteristics of Trialled Subjects

Characteristics	Subjects (*N* = 21)
Gender	
Female	19 (90.5%)
Male	2 (9.5%)
Age (years) at enrollment	45.4 ± 18.5
Years since diagnosis	9.7 ± 7.3
Ethnicity	
Non‐Hispanic/Latino	21 (100.0%)
Race	
African American	1 (4.3%)
Caucasian	20 (95.7%)
Previous surgery	12 (57.1%)
Baseline VAS score	7.9 ± 0.3

Data presented as mean ± SD (SEM for VAS) or *n* (%).

SD, standard deviation; SEM, standard error of the mean.

Figure 1B shows the study design and patient flow. Two subjects withdrew consent before the completion of the trial portion of the study, and of the 21 subjects who completed trial stimulation, 17 (81%) reported ≥ 40% pain relief (average pain relief: 78%) and were deemed to have had successful trials. Of the 4 subjects who did not have successful trials, 3 did not experience adequate pain relief, and 1 was unable to tolerate the temporary implanted leads and withdrew from the study. Of the 17 subjects who had successful trial stimulations, 3 withdrew consent before receiving permanent implants, and another had the implant excised after 1 month due to an incision site infection. In total, 13 subjects received permanent implants and completed all follow‐up assessments through the end of the 12‐month study period.

### Safety

No neurological deficits were observed during the study period in the subjects who received permanently implanted pulse generators (IPGs) or who were trialed with temporary devices, and a total of 10 study‐related AEs were recorded by study investigators. These included 4 device‐related AEs, 5 AEs related to the implantation procedure, and 1 stimulation‐related AE. All AEs were resolved without sequelae by the end of the study period (Table 2).

**Table 2:** Treatment Related Adverse Events (AEs)

Type of AE	Number of Subjects (*n*)
Device related	
Device dislocation (*n* = 2)	2
Implant site pain (*n *= 1)	1
Medical device discomfort (*n* = 1)	1
Stimulation related	
Lightheadedness (*n* = 1)	1
Procedure related	
Incision site infection (*n* = 1)	1
Medical devise site pain (*n* = 1)	1
Device dislocation (*n* = 1)	1
Rash related to adhesive strips (*n* = 1)	1
Patient did not tolerate leads in epidural space	1

### Pain Outcomes

**Figure 2:**
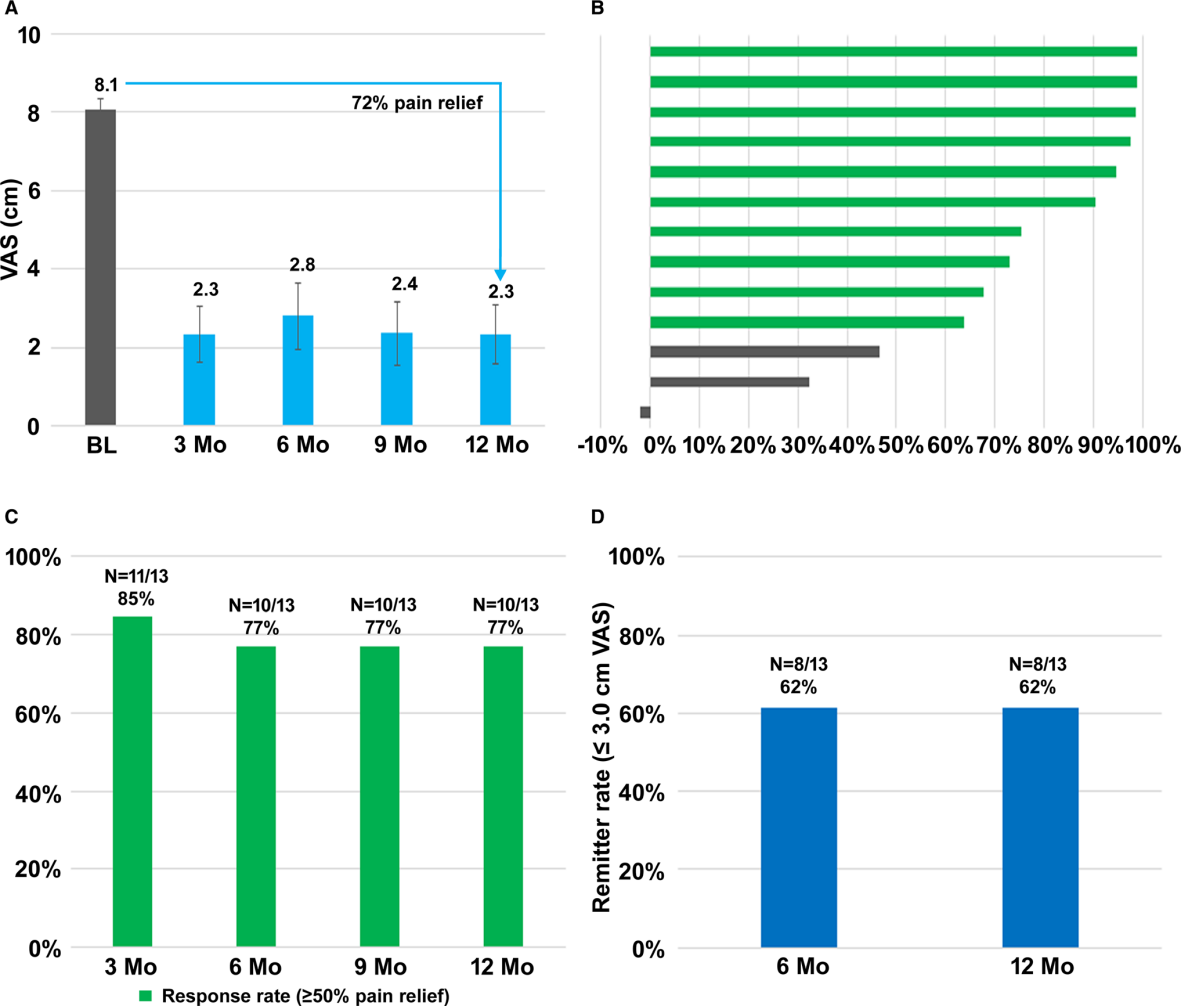
Stimulation with 10‐kHz spinal cord stimulation is associated with pain relief in subjects with permanent implants (*n* = 13). A, Mean VAS scores ± SEM. B, Pelvic pain relief in individual subjects after 12 months of stimulation. C, Responder rate after 3, 6, 9, and 12 months of stimulation. D, Pain remission rates after 6 and 12 months of stimulation.

Mean VAS scores of patients who received permanent implants decreased from 8.1 cm at baseline to 2.3 cm after 3 months of 10‐kHz SCS, as shown in Figure 2A. This decrease was maintained through 12 months of stimulation and corresponds to a 72% decrease in VAS scores. After 3 months of stimulation, 10 of 13 subjects (77%) were responders, defined as those who reported pain relief of ≥50%, and these responses lasted through the 12‐month follow‐up visit (Figure 2B,C). The proportion of pain remitters, those who reported VAS scores of ≤3.0 cm, after both 6 months and 12 months of 10‐kHz SCS was 8 of 13 subjects (62%; Figure 2D).

**Figure 3:**
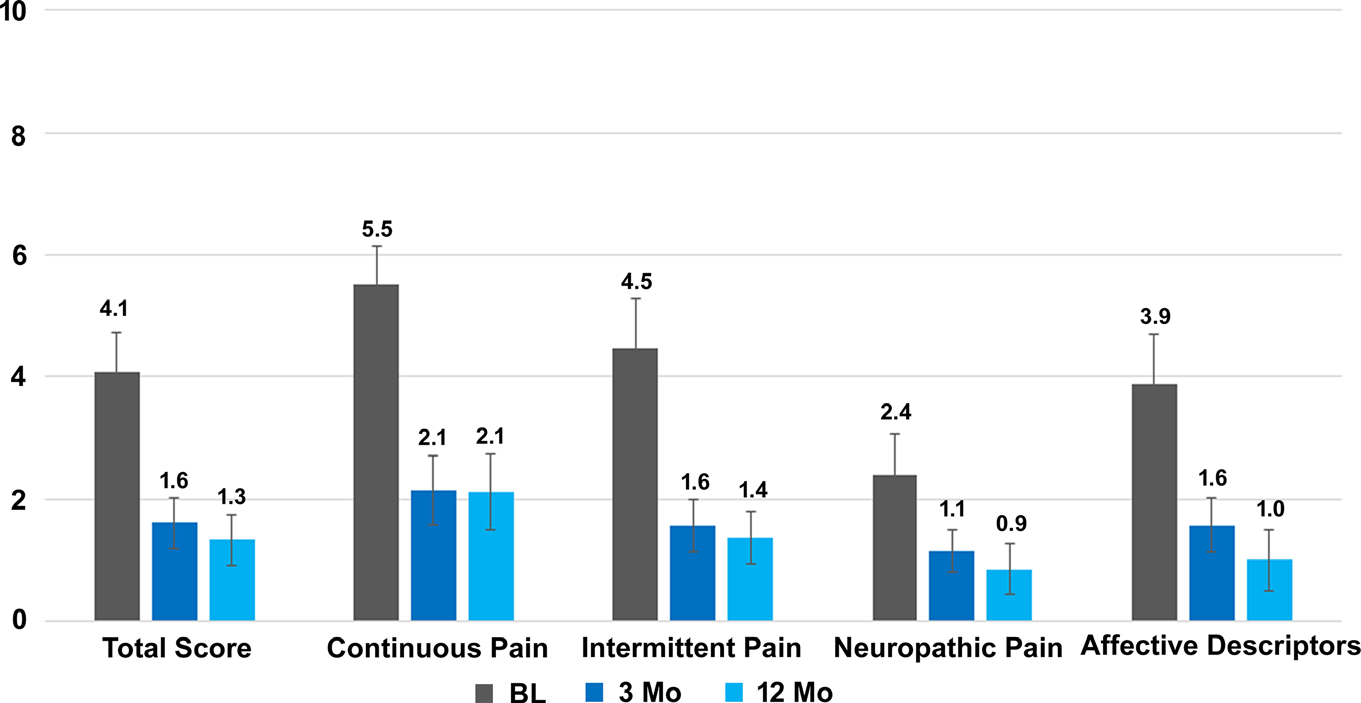
Mean short‐form McGill Pain Questionnaire 2 scores decreased after the initiation of 10‐kHz spinal cord stimulation. Values are means ± SEM.

Mean SF‐MPQ‐2 scores also decreased from baseline levels after 3 months of 10‐kHz SCS, and these decreases were observed in both overall scores as well as in all 4 subdomains including affective descriptors of pain, as shown in Figure 3. The improvement in mean scores for all items was maintained through the 12‐month study period.

### Disability and Functioning

**Figure 4:**
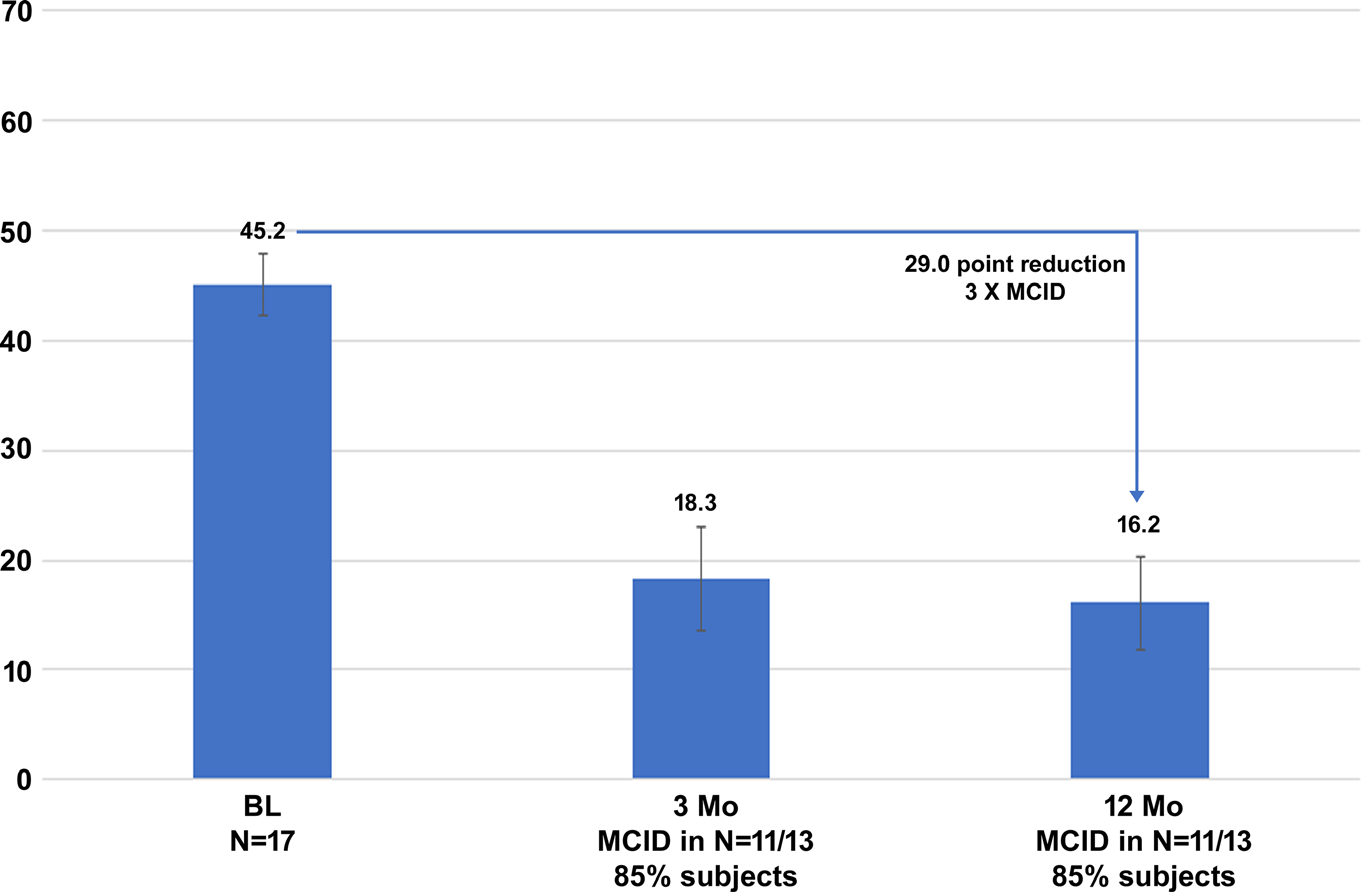
Disability scores decreased after the initiation of 10‐kHz SCS. The 29.0‐point reduction in Pain Disability Index score from baseline to 12 months of stimulation is 3‐fold greater than the minimal clinically important difference (MCID). The MCID was met or exceeded in 85% of subjects after 3 and 12 months of stimulation. BL, Baseline.

**Figure 5:**
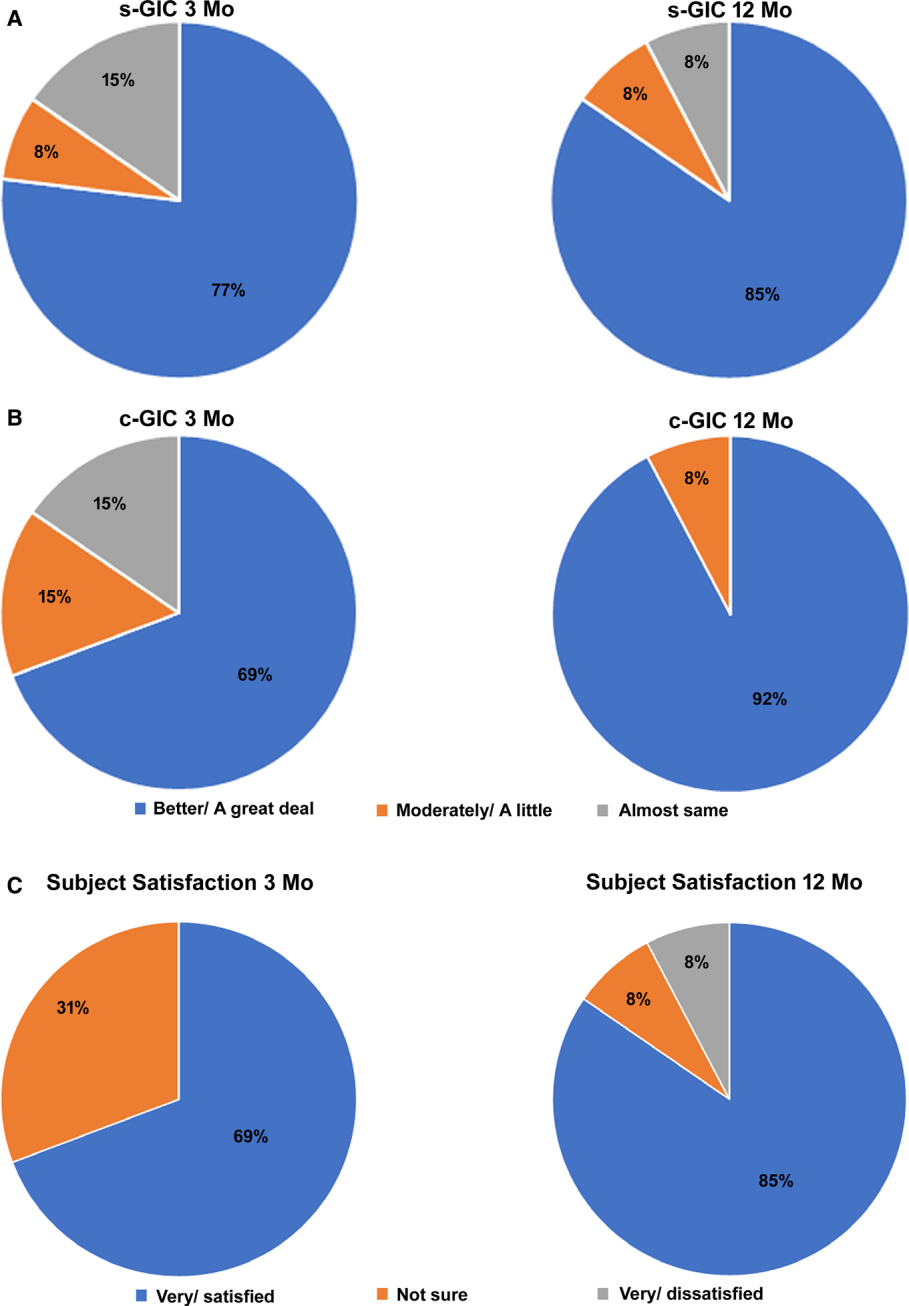
Most subjects (A) and clinicians (B) reported their symptoms were “better” or “a great deal better” on the Global Impression of Change (GIC) scale. The responses from both groups were more positive after 12 months of stimulation than after 3 months. (C) Most subjects were satisfied with 10‐kHz SCS treatment after 3 months, and this proportion increased after 12 months of stimulation.

Improvement in subject disability was assessed using PDI scores, and these results are shown in Figure 4. Mean PDI scores decreased by 29.0 points after 12 months of 10‐kHz SCS, and the magnitude of decrease was clinically meaningful in 11 of 13 subjects (85%). Assessment of subject functioning using the GIC scale showed that most subjects and clinicians perceived functioning to be “better” or “a great deal better” after 3 months of 10‐kHz SCS, and that number increased after 12 months of stimulation, as shown in Figure 5A,B.

### Subject Satisfaction

The study subjects were surveyed about their satisfaction with 10‐kHz SCS treatment at follow‐up visits. After 3 months of stimulation, 69% of subjects reported being satisfied or very satisfied with treatment, and this proportion increased to 85% after 12 months of treatment, as shown in Figure 5C.

## Discussion

Currently, CPP is treated with a combination of medication, physical therapy, nerve blocks, surgery, and neuromodulation. Many of these patients, however, do not obtain long‐term benefit from these treatment modalities, and there is an unmet need for new therapies in this population.^6^ Although conventional SCS has been used to treat CPP, results are inconsistent and appropriate lead placement is difficult due to the innervation patterns of the pelvis.^2^, ^10^ A retrospective case series of patients with CPP who were treated with conventional SCS found 74% pain relief in these patients,^9^ but a review of data from a single institution showed that patients who received conventional SCS to treat pelvic or abdominal pain had the highest rate of complications (70%) and the highest rate of explant procedures among all types of pain included in the sample, often due to loss of therapeutic effect.^19^

The evidence for the efficacy of 10‐kHz SCS relieving chronic pain in multiple contexts, improving patients’ quality of life, and reducing opioid consumption,^20^, ^21^ and its efficacy in treating CPP is supported by a small case series.^13^ Unlike SCS at lower frequencies, animal models suggest 10‐kHz SCS selectively activates inhibitory interneurons in the spinal dorsal horn at low intensities below the threshold activating the dorsal column.^22^ This selectivity at low intensities suggests the mechanism of action by which 10‐kHz SCS is able to deliver paresthesia‐independent pain relief. The results presented here further support the safety and efficacy of 10‐kHz SCS in subjects with pelvic pain. No neurological deficits were observed during the 12‐month stimulation period compared to baseline measures, none of the observed AEs were serious, and all were resolved without sequelae.

Mean VAS scores declined within 3 months of treatment, and these pain decreases were durable through the entire 12‐month study. Mean pain relief in subjects with permanent implants (per‐protocol population) was 72% after 12 months of stimulation, a result that is comparable to other published studies of 10‐kHz SCS for treating chronic pain. The randomized, controlled SENZA‐RCT showed 67% pain relief for low back pain and 70% for leg pain,^11^ and Amirdelfan et al.^14^ reported pain relief of up to 79% for neck pain and 86% for upper limb pain with the use of 10‐kHz SCS. Likewise, a prospective study of 10‐kHz SCS in subjects with chronic abdominal pain reported mean 74% mean pain relief,^23^ and a retrospective review of data from real‐world patients with chronic limb and trunk pain found an average of 63% pain relief.^24^

Mean SF‐MPQ‐2 scores at 3 and 12 months for pelvic pain decreased overall as well as in all 4 subdomains: continuous, intermittent, and neuropathic pain, and affective descriptors. These results are comparable to those in prospective studies in subjects with upper limb and neck pain^14^ and abdominal pain,^23^ which also found decreases in total and in all 4 subdomains of pain comprising the SF‐MPQ instrument, and these decreases persisted for at least 12 months of treatment.

The current results also show that 77% of per‐protocol population were responders (pain relief ≥ 50%) after 12 months of stimulation and 62% of per‐protocol population were pain remitters (VAS score ≤ 3.0 cm). Comparable published values for response rates after 12 months of 10‐kHz SCS include 79% for back and leg pain from the SENZA‐RCT^11^ and 89% to 95% in subjects with neck and upper limb pain.^14^ Remitter rates from these sources were 67% to 69% in SENZA‐RCT and 78% to 80% for neck and upper limb pain.

In addition to reduced pain, treatment with 10‐kHz SCS reduced mean PDI scores by 29 points in these subjects with CPP, indicating improved functioning. The magnitude of this decrease is notable, as it is 3‐fold larger than the minimal clinically important difference established by Soer et al.^25^ for patients with musculoskeletal and spinal disorders. This objective measure of subject functioning was supported by results of subject‐ and clinician‐reported GIC, which showed a strong subjective assessment of improvement.

The interpretation of these results is limited by factors including small cohort size and lack of randomization with a control arm. Because the main objective of this pilot study was to test the safety and efficacy of 10‐kHz SCS in this patient population with unmet need, the study was designed as a single arm. The findings from this study would encourage larger randomized study with appropriate controls. Further studies, including retrospective studies of real‐world data, could help to further define the potential benefits and risks of 10‐kHz SCS in patients with CPP.

## Conclusions

The data reported here show that 10‐kHz SCS is a safe and effective treatment option for treating symptoms of CPP. Most subjects tested responded to stimulation with decreased pain levels and increased daily functioning with duration of benefits lasting through the end of the 12‐month study period. These responses are consistent with results of 10‐kHz SCS treatment in other forms of chronic pain.

## Funding

This study was sponsored by Nevro Corp.

## Conflicts of Interest

J.T. and S.L. are consultants of Nevro Corp. A.R. and J.S. are employees of Nevro Corp. T.S. has no conflicts of interest to disclose.

## Author Contributions

J.S. was responsible for conception and design planning, conduct, data analysis, and interpretation. J.T., T.S., and S.L. were involved in conduct, reporting, acquisition of data, and interpretation of data. A.R. was responsible for data analysis, interpretation, and preparation of the outline for the manuscript, and drafted the manuscript with the help of an external medical writer. All authors reviewed and approved the final manuscript.

## Supporting information


**Table S1.** Inclusion criteria.
**Table S2.** Exclusion criteria.
**Figure S1.** Etiologies of subjects included in the study.
