# Supplementary material for: A Prospective, Multi‐Center, Clinical Trial of a 10‐kHz Spinal Cord Stimulation System in the Treatment of Chronic Pelvic Pain
Source: Pain Pract. 2020 Aug 8;21(1):45–53. doi: 10.1111/papr.12932 (PMC7818476; doi:10.1111/papr.12932)
Supplement: Supplementary file 1 — Table S1. Inclusion criteria. Table S2. Exclusion criteria. Figure S1. Etiologies of subjects included in the study. [file PAPR-21-45-s001.docx]

**Supplementary Table S1. Inclusion criteria**

| Have been clinically diagnosed with chronic pelvic pain (pain that occurs in the region of the pelvis), including diagnoses such as, but not limited to, complex regional pain syndrome (CRPS types 1 and 2), interstitial cystitis/ painful bladder syndrome, post-hysterectomy pain, post-prostatectomy pain, vulvodynia, chronic ovarian pain, and pudendal neuralgia. |
| --- |
| Have been refractory to conservative therapy for a minimum of 3 months, including assessment of at least 2 different classes of medications and/or an anesthetic block as clinically appropriate. |
| Average pain intensity (over the last 7 days) of ≥5 out of 10 cm on the Visual Analog Scale (VAS) in the primary area of pain at enrollment. |
| Be on stable pain medications, as determined by the Investigator, for at least 28 days prior to assessing pain intensity as described in inclusion criterion #3, and be willing to stay on those medications with no dose adjustments until activation of the permanently implanted SCS device. |
| Have stable neurological status measured by motor, sensory and reflex function as determined by the investigator. |
| Be 18 years of age or older at the time of enrollment. |
| Be an appropriate candidate for the surgical and implant procedures required in this study based on the clinical judgment of the implanting physician. |
| Be able to understand written and spoken English. |
| Be capable of subjective evaluation; patient must be able to describe and rate his/her pain levels. |
| Be willing and capable of giving informed consent. |
| Be willing and able to comply with study-related requirements, procedures, and visits. |
| Have adequate cognitive ability to use a patient programmer and recharger as determined by the Investigator. |
| Be an appropriate candidate based on the psychological evaluation and the discretion of the investigator. |

**Supplementary Table S2. Exclusion criteria**

| Have a medical condition or pain in other area(s) not intended to be treated with spinal cord stimulation (SCS) that could interfere with study procedures, accurate pain reporting, and/or confound evaluation of study endpoints, as determined by the Investigator (such as arthritis or bursitis of the hip or fibromyalgia). |
| --- |
| Have a current diagnosis of a progressive neurological disease such a multiple sclerosis, chronic inflammatory demyelinating polyneuropathy, rapidly progressive arachnoiditis, brain or spinal cord tumor, central deafferentation syndrome, acute herniating disc, severe spinal stenosis and brachial plexus injury, as determined by the Investigator. |
| Have a current diagnosis or condition such as a coagulation disorder, bleeding diathesis, platelet dysfunction, progressive peripheral vascular disease or uncontrolled diabetes mellitus that presents excess risk for performing the procedure as determined clinically by the investigator. |
| Have had prior use of a neuromodulation device. |
| Diagnosed with Crohn’s Disease, Irritable Bowel Syndrome, ulcerous colitis, endometriosis or any other inflammatory disease that is ongoing. |
| Have used botulinum toxin within the prior 6 months to treat pelvic pain symptoms (use of botulinum toxin greater than 6 months prior to enrollment is allowed). |
| Be benefitting from an interventional procedure and/or surgery to treat chronic pelvic pain (Subjects should be enrolled at least 30 days from last benefit). |
| Have an existing drug pump and/or another active implantable device that would be contraindicated for use with the Senza System, based on device labeling. |
| Have a condition currently requiring or likely to require the use of MRI in a manner contraindicated with the Senza System as defined in the device labeling. |
| Have a condition currently requiring or likely to require diathermy. |
| Have metastatic malignant disease or active local malignant disease. |
| Have a life expectancy of less than 1 year. |
| Have an active systemic infection or a local infection at the anticipated surgical entry sites. |
| Be pregnant or nursing. Patients of child-bearing potential must use reliable forms of contraception. |
| Be immunocompromised. |
| Have been known to be allergic or have shown hypersensitivity to any materials of the neurostimulation system which come in contact with the body. |
| Be concomitantly participating in another clinical study. |
| Be involved in an injury claim under current litigation. |
| Have an active or unsettled worker’s compensation claim. |

**Supplementary Figure S1.** Etiologies of subjects included in the study*


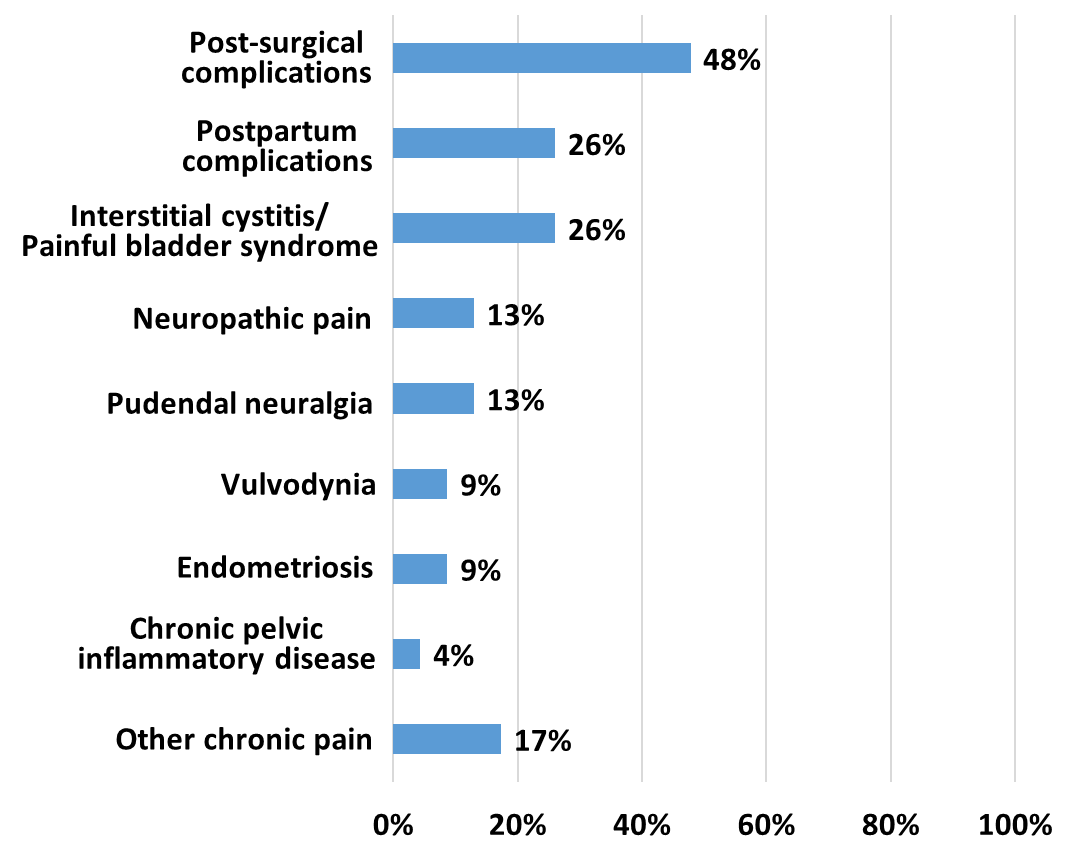


*some subjects had multiple pain etiologies
